# Reduced Levels of Plasma Kisspeptin During the Antenatal Booking Visit Are Associated With Increased Risk of Miscarriage

**DOI:** 10.1210/jc.2014-1953

**Published:** 2014-08-15

**Authors:** C. N. Jayasena, A. Abbara, C. Izzi-Engbeaya, A. N. Comninos, R. A. Harvey, J. Gonzalez Maffe, Z. Sarang, Z. Ganiyu-Dada, A. I. Padilha, M. Dhanjal, C. Williamson, L. Regan, M. A. Ghatei, S. R. Bloom, W. S. Dhillo

**Affiliations:** Section of Investigative Medicine (C.N.J., A.A., A.I.-E., A.N.C., Z.S., Z.G.-D., M.A.G., S.R.B., W.S.D.) and Imperial Clinical Trials Unit (H.G.M.), Imperial College London, London W12 ONN, United Kingdom; Medical Oncology Laboratory (R.A.H., A.I.P.), Charing Cross Hospital Campus, Imperial College National Health Service Healthcare Trust, London W6 8RF, United Kingdom; Department of Obstetrics and Gynaecology (M.D.), Queen Charlotte's Hospital, Imperial College National Health Service Healthcare Trust, London W12 0HS, United Kingdom; Department Obstetrics and Gynaecology (C.W.), King's College London, London SE5 9PJ, United Kingdom; and Department of Obstetrics and Gynaecology (L.R.), St Mary's Hospital, Imperial College National Health Service Healthcare Trust, London W2 1NY, United Kingdom

## Abstract

**Context:**

Kisspeptin is a recently identified hormone encoded by the *KISS1* gene, playing a critical role in human reproduction. Plasma kisspeptin levels rise dramatically during normal pregnancy due to placental synthesis, which implicates it as a potential tool for assessing risks of pregnancy complications. No previous prospective study has investigated the association between plasma kisspeptin and risk of miscarriage.

**Objective:**

The objective of the study was to determine whether a single plasma kisspeptin or serum human chorionic gonadotropin (hCG) measurement in asymptomatic women attending their booking antenatal visit is associated with miscarriage.

**Design:**

This was a prospective cohort study.

**Setting:**

The study was conducted at a tertiary obstetric center.

**Participants:**

A total of 993 asymptomatic pregnant women with a gestation of 6 weeks or longer attending routine antenatal booking visit were recruited between January 2010 and December 2012.

**Main Outcome Measures:**

Plasma kisspeptin and serum hCG were measured during the antenatal booking visit. Pregnancy outcome was recorded prospectively.

**Results:**

Plasma kisspeptin correlated with gestation (r^2^ = 0.57; *P* < .0001). Gestational age-corrected (multiples of median) plasma kisspeptin was 60.4% lower (*P* < .001), and multiples of median-hCG was 36.1% lower (*P* < .001) in women later diagnosed with miscarriage compared with women without miscarriage. Increased plasma kisspeptin was associated with reduced miscarriage risk, even after adjusting for age, body mass index, gestational age, smoking, and blood pressure [odds ratio 0.13 (95% confidence interval 0.08–0.22), *P* = .0001]. Kisspeptin had a higher diagnostic performance for miscarriage than hCG (receiver-operator characteristic-area under the curve 0.899 ± 0.025 plasma kisspeptin; 0.775 ± 0.040, serum hCG, *P* < .01 vs plasma kisspeptin).

**Conclusion:**

Our data suggest for the first time that a single plasma kisspeptin measurement taken during the antenatal booking visit provides a potential novel marker for identifying asymptomatic pregnant women at a gestation of 6 weeks or greater at increased risk of miscarriage.

Miscarriage is defined as spontaneous pregnancy loss prior to 24 weeks of gestation (1). Ten to 20% of all clinically recognized pregnancies end in miscarriage (2, 3). Women affected by a single miscarriage not only suffer devastating emotional consequences (4) but are also at increased risk of developing serious antenatal morbidities such as preeclampsia and preterm delivery during subsequent pregnancies (5–7). Prior to 6 weeks' gestation, most miscarriages result from cytogenetic abnormalities in the embryo such as chromosomal trisomy (8). However, later during gestation, other causes of miscarriage, such as placental insufficiency, intrauterine infection, and thrombosis, become more common. There are currently no proven treatments to prevent noncytogenetic causes of miscarriage; however, this may reflect that there is currently no established marker to identify women at increased risk of miscarriage. Fetal viability may be estimated using serial measurement of serum human chorionic gonadotropin (hCG) during multiple hospital visits; however, approximately 20% of cases resulting in miscarriage are also associated with rising levels of serum hCG, which are typical of viable pregnancy, and the clinical utility is limited (9). There is therefore both a delay and a high degree of uncertainty in diagnosing miscarriage using this approach, which can be a source of further distress for affected couples.

Abnormal placentation (placental development) is found in two-thirds of cases of miscarriage (10, 11). The recently identified hormone kisspeptin has been suggested to play an important regulatory role in placentation. Kisspeptin consists of a group of arginine-phenylalanine (RF) amide peptides encoded by the *KISS1* gene, which bind to the kisspeptin receptor (12–15). Kisspeptin is expressed most abundantly in the syncytiotrophoblast cells of the placenta, in which it may regulate invasion into the maternal uterine wall (14, 16, 17). Kisspeptin is also expressed in areas of the brain such as hypothalamus, amygdala, and caudate nucleus in addition to the pituitary, pancreas, adipose, testis, lymphocytes, and spleen (13, 15). Circulating levels of kisspeptin increase gradually during pregnancy, becoming markedly elevated up to 7000-fold during later pregnancy when compared with nonpregnant women (18). Recent case-control studies have suggested that circulating kisspeptin levels are reduced in women with preeclampsia and intrauterine growth retardation, when compared with uncomplicated pregnancies (19, 20). Levels of placental kisspeptin expression are significantly lower in women with recurrent miscarriage when compared with placental tissue in electively terminated pregnancies (21). This preliminary evidence therefore suggests that low placental kisspeptin levels may be associated with increased risk of miscarriage or other serious obstetric complications if the fetus survives. It is currently not known whether plasma kisspeptin levels during pregnancy are associated with miscarriage.

We conducted a prospective cohort study including more than 900 women at a single obstetric center to determine whether levels of plasma kisspeptin or serum hCG are associated with miscarriage in asymptomatic women at a gestation of 6 weeks or longer attending their booking antenatal visit.

## Materials and Methods

This study was performed in accordance with the Declaration of Helsinki following ethical approval (West London Research Ethics Committee; Q0406/80). Women attending routine antenatal booking visit at Queen Charlotte's and Chelsea Hospital London (United Kingdom) were invited to participate between January 2010 and December 2012. Nine hundred and ninety-three pregnant women attending their routine antenatal booking clinic appointment were recruited. After written informed consent, a single blood sample was taken for the measurement of plasma kisspeptin and serum hCG. Women with renal failure were excluded due to assay interference with kisspeptin measurement. Emergency admissions or cases of suspected miscarriage were excluded. Twelve women were lost to follow-up, despite attempts by obstetric staff to contact these women. The remaining 981 women were followed up until the outcome of pregnancy was known.

### Patient details and outcomes

All pregnancies were dated according to a dating ultrasound scan (typically at 8–14 wk gestation) performed in the antenatal clinic. Ultrasound parameters from a 20-week fetal anomaly scan, patient-held clinical notes, and hospital electronic records were collated to establish whether miscarriage or live birth had occurred. Miscarriage was diagnosed using ultrasound scanning or was self-reported. Patient details are summarized in Table 1.

**Table 1.** Clinical Characteristics of Study Participants

	n	Mean Age, y (±SD)	Mean BMI, kg/m^2^ (±SD)	Mean Parity (±SD)	Mean Gestation, wk (±SD)	Smoking During Pregnancy, %	Mean Systolic BP, mm Hg	Mean Diastolic BP, mm Hg
Total number of women participating in study	981	32.6 (±2.0)	24.8 (±5.5)	0.6 (±0.8)	11.2 (±2.0)	4.0	107.9 (±17)	65.5 (±8.8)
Number of women with singleton pregnancy without miscarriage	899	32.4 (±5.1)	24.7 (±5.4)	0.7 (±0.8)	11.3 (±1.9)	4.0	107.8 (±17.2)	65.5 (±8.9)
Number of women with singleton miscarriage	50	33.1 (±4.8)	26.3 (±7.4)	0.6 (±0.8)	9.8 (±3.1)	6.2	108.2 (±12.7)	65.6 (±8.4)
Number of women with multiple pregnancy	32	35.5 (±5.6)	25.9 (±5.6)	0.5 (±0.9)	10.7 (±1.5)	3.4	109.9 (±12.9)	68.1 (±8.5)
Ethnicity (n = 981)								
Caucasian	574 (58.5%)							
South Asian	121 (12.3%)							
Afrocaribbean	107 (10.9%)							
Other	109 (11.1%)							
Mixed	24 (2.4%)							
Unknown	46 (4.7%)							

### Measurement of plasma kisspeptin immunoreactivity

Measurement of plasma kisspeptin immunoreactivity was performed using an established in-house RIA previously developed in our laboratory (22, 23). In brief, a known quantity of I-125 labeled kisspeptin-54 peptide is used to compete with an unknown quantity of kisspeptin for binding to a limited quantity of antibody derived from sheep antiserum raised against kisspeptin. Radioactivity of charcoal-separated antibody-peptide complexes is inversely proportional to levels of kisspeptin in the sample. The antibody cross-reacted 100% with human kisspeptin-54, kisspeptin-14, and kisspeptin-10 and less than 0.01% with other related RF amide proteins, including prolactin-releasing peptide, RF amide-related peptide (RFRP) 1, RFRP2, RFRP3, pyroglutamylated RF amide peptide 43, neuropeptide FF, and neuropeptide AF. The limit of detectability was 2 pmol/L, and the intra- and interassay coefficients of variation were 8.3% and 10.2%, respectively.

### Measurement of total hCG immunoreactivity

Measurement of plasma hCG was performed using the Siemens Immulite 2000 total immunometric hCG assay run on board the Siemens Immulite 2000 instrument (Siemens Healthcare Diagnostics). According to the manufacturer's kit insert, the analytical sensitivity of the assay is 0.4 IU/L. Performance is monitored daily and internal quality control samples ranging from 5 to 250 IU/L yield intra- and interassay coefficients of variation of less than 10%. Studies using the latest World Health Organization standard preparations for common hCG protein variants have demonstrated that the Immulite 2000 assay detects all hCG isoforms, but is associated with a relative slight overestimation of hCGβ and an underdetection of the hCGβ core fragment isoforms (24, 25).

### Data analysis and statistics

Sample size aimed to recruit sufficient cases to determine the association of plasma kisspeptin with miscarriage, correcting for confounding variables that may confer an increased risk of miscarriage [ie, participant age, gestational age, body mass index (BMI), smoking, diastolic blood pressure (BP), and systolic BP]. Data was analyzed using Prism 5 (GraphPad). To account for the variation in circulating kisspeptin and hCG levels during pregnancy, we calculated multiple of median (MoM) levels of each hormone measurement during the gestation when blood was collected. Data are presented as mean ± SD. Pairs of means were analyzed using the unpaired two-tailed *t* test. Multiple means were compared using one-way ANOVA with Tukey's multiple comparison test. Logistic regression was used to analyze associations between plasma kisspeptin and hCG with the risk of miscarriage; the rationale for log transformation was to reduce the variability in kisspeptin levels (particularly at high levels) to better study the association with risk of miscarriage. *P* < .05 was considered statistically significant.

## Results

### Baseline characteristics of pregnant subjects attending their antenatal booking visit

Nine hundred ninety-three women attending antenatal clinic for a routine booking antenatal appointment were recruited to the study. Twelve women (ie, 1% of the sample) were lost to follow-up, so their data were excluded from all analyses. The remaining 981 women had a mean gestation of 11.2 weeks (range 5.9–22.1 wk) (see Table 1 for characteristics). Thirty-two pregnancies were multiple (30 twin pregnancies of which six were monochorionic; two triplet pregnancies). No participants were attending as an emergency or presented with symptoms of threatened miscarriage such as abdominal pain or vaginal bleeding.

### Distribution of plasma levels of kisspeptin in pregnant women at the antenatal booking visit with a singleton pregnancy not resulting in miscarriage

**Figure 1.**
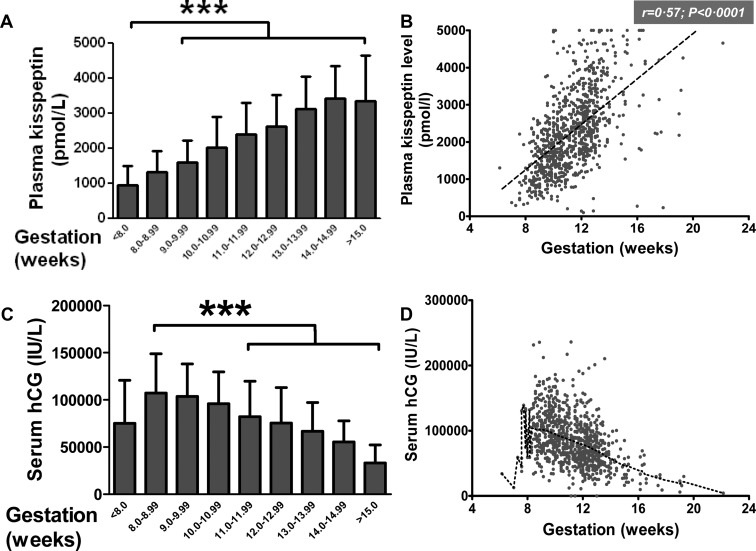
Plasma kisspeptin and serum hCG have distinct distributions when classified by gestation in women with singleton pregnancy who did not miscarry. A and B, In singleton pregnancy not resulting in miscarriage, the mean plasma levels of kisspeptin during the antenatal booking visit increased progressively with increasing gestation (A) and correlated significantly with gestational age (B). C and D, In singleton pregnancy not resulting in miscarriage, the mean serum hCG during the antenatal booking visit peaked at approximately 8 weeks' gestation and then decreased progressively with increasing gestation (C). Scatter plot of serum hCG vs gestational age is shown with a coarse Lowess plot (dotted line), denoting the overall trend in their relationship (D). Data are mean ± SD. ***, *P* < .0001.

In single pregnancies not resulting in miscarriage, levels of plasma kisspeptin increased steadily during each week of gestation during the first trimester of pregnancy (Figure 1A). Plasma kisspeptin in singleton pregnancies was highly significantly and positively correlated with the gestational week of pregnancy (r = 0.57; *P* < .0001) (Figure 1B). Serum hCG in singleton pregnancies had a complex relationship with gestational age; levels peaked around the eighth gestational week before reducing with increasing gestational week (Figure 1, C and D). Log transformation of serum hCG in singleton pregnancies suggested a significant and negative correlation with gestational age [r = −0.41, 95% confidence interval (CI) −0.47 to −0.36] (data not shown).

In singleton pregnancies not resulting in miscarriage, MoM plasma kisspeptin levels were classified according to the duration of sample storage at −20°C prior to kisspeptin measurement (between 10 and 31 months; Supplemental Figure 1). No significant differences between MoM kisspeptin levels among any group were observed (*P* = .07).

### Relationship between plasma kisspeptin and miscarriage in singleton pregnancies

**Figure 2.**
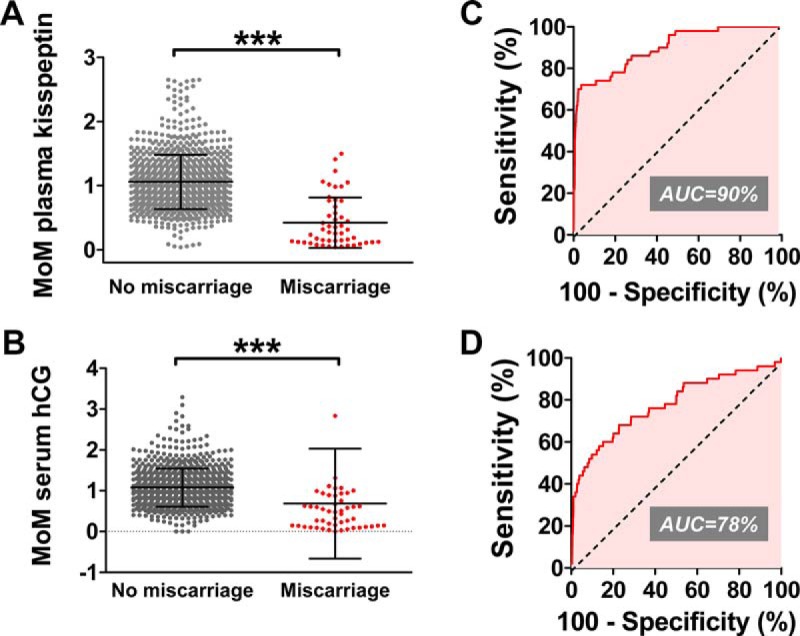
Plasma kisspeptin and serum hCG as markers of miscarriage in asymptomatic women during singleton pregnancy. A and B, MoM levels of plasma kisspeptin (A) and serum hCG (B) were calculated to correct for gestational age at the time of the blood collection in asymptomatic women attending their antenatal booking visit for a singleton pregnancy. Levels of each hormone are presented in asymptomatic women who were and were not later diagnosed with miscarriage. C and D, ROC analysis of diagnostic performances of plasma kisspeptin (C) and serum hCG with respect to miscarriage. Data are mean ± SD. ***, *P* < .0001. AUC, area under the curve.

Fifty of the 981 women attending antenatal clinic with a singleton pregnancy (5.1%) were diagnosed with miscarriage after recruitment to the study (Supplemental Figure 2). MoM (gestation corrected) kisspeptin levels were 60% lower in women with a singleton pregnancy who later experienced miscarriage when compared with unaffected pregnancies (MoM kisspeptin: 1.06 ± 0.42, singleton, no miscarriage; 0.42 ± 0.39, singleton with miscarriage, *P* < .001 vs singleton, no miscarriage) (Figure 2A). By comparison, MoM hCG levels were only 36% lower in singleton pregnancies during miscarriage when compared with singleton pregnancies, which did not result in miscarriage (MoM kisspeptin: 1.08 ± 0.47, singleton; no miscarriage, 0.69 ± 1.35, singleton with miscarriage, *P* < .001 vs singleton, no miscarriage) (Figure 2B). Receiver-operator characteristic (ROC) curve analysis suggested that kisspeptin had a higher diagnostic performance with respect to miscarriage when compared with hCG (ROC area under curve: 0.899 ± 0.025, kisspeptin; 0.775 ± 0.040, hCG, *P* < .01 vs kisspeptin) (Figure 2, C and D). A kisspeptin threshold of 1630 pmol/L had 86% sensitivity (95% CI 73%–94%) and 70% specificity (95% CI 67%–73%) for detecting miscarriage, and a kisspeptin threshold of 1463 pmol/L had an 80% sensitivity (95% CI 66%–90%) and a 75% specificity (95% CI 72%–78%) for miscarriage. Patients with kisspeptin levels less than 1630 pmol/L had a 13.8% chance of miscarriage being subsequently diagnosed (ie, 13.8% positive predictive value), whereas patients with kisspeptin levels greater than 1630 pmol/L had a 1.0% chance of miscarriage being subsequently diagnosed (ie, 99.0% negative predictive value).

Kisspeptin increased to a significantly lesser extent with gestational age in the cohort of women who experienced miscarriage when compared with the cohort of women with uncomplicated pregnancy [slope coefficient for increase in plasma kisspeptin (picomoles per liter per week): 305 ± 15, singleton without miscarriage; 94 ± 30, singleton with miscarriage; F = 25.2, DFn = 1, DFd = 945, *P* < .0001] (data not shown). A logistic regression analysis was performed to investigate whether the relationship between kisspeptin and miscarriage was independent of known confounders (Table 2). The unadjusted model revealed that the odds of experiencing a miscarriage decreased by 89% [odds ratio (OR) 0.11; 95% CI 0.07–0.17; *P* < .0001] for each 10-fold increase in kisspeptin. After adjustment for subject age, gestation, smoking, BP, and BMI, the odds of experiencing miscarriage decreased by 87% (OR 0.13; 95% CI 0.08–0.22; *P* < .0001) for each 10-fold increase in kisspeptin.

**Table 2.** Logistic Regression Model for Miscarriage Risk in Relation to Plasma Kisspeptin in Women During the Antenatal Booking Visit

Parameter	Unadjusted Model 1	Adjusted	
		Model 2	Model 3
(Log) Kisspeptin	0.11 (0.07, 0.17) *P* < .0001	0.14 (0.08, 0.22) *P* < .0001	0.13 (0.08, 0.22) *P* < .0001
Age		1.02 (0.94, 1.11) *P* = .627	1.03 (0.94, 1.12) *P* = .565
BMI		0.96 (0.88, 1.05) *P* = .406	0.97 (0.89, 1.07) *P* = .559
Gestational age		0.72 (0.55, 0.95) *P* = .019	0.68 (0.51, 0.91) *P* = .010
Smoking		1.31 (0.24, 7.23) *P* = .755	1.23 (0.22, 6.78) *P* = .811
Diastolic BP			0.99 (0.93, 1.05) *P* = .649
Systolic BP			0.99 (0.96, 1.03) *P* = .771

The unadjusted odds of experiencing a miscarriage decrease by 89% (OR 0.11; 95% CI 0.07–0.17; *P* = .0001) for each unit increase in (log) plasma kisspeptin. After adjusting the model by age, gestational age (weeks), and BMI, we found that the effects of kisspeptin change slightly. After adjustment for confounders, the odds of experiencing a miscarriage still decrease by 87% (OR 0.13; 95% CI 0.08–0.22; *P* = .0001) for each unit increase in (log) kisspeptin.

### Combined testing of kisspeptin with hCG

A logistic regression analysis was performed to explore whether the diagnostic accuracy of using combined kisspeptin and hCG levels was superior to kisspeptin alone. Combined kisspeptin and hCG measurement (OR 0.10; 95% CI 0.06–0.17; *P* < .0001) had similar diagnostic accuracy when compared with kisspeptin measurement alone (OR 0.11; 95% CI 0.07–0.17; *P* < .0001).

### Relationship of plasma kisspeptin with time to diagnosis of miscarriage

**Figure 3.**
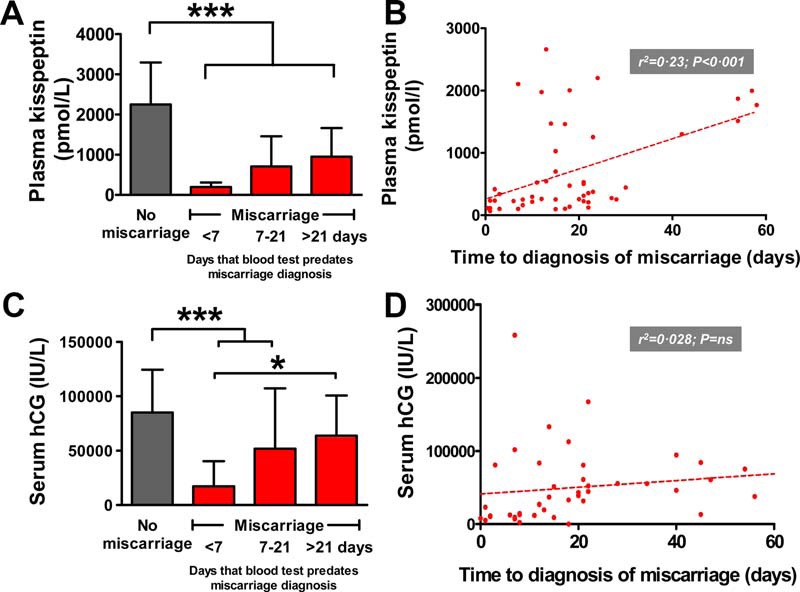
Time elapsed after the measurement of plasma kisspeptin or serum hCG to the diagnosis of miscarriage. A, Plasma kisspeptin is significantly lower in pregnancies during miscarriage, regardless of the time elapsed between the blood test and the diagnosis of miscarriage. B, Levels of plasma kisspeptin correlated with time elapsed until diagnosis of miscarriage in asymptomatic singleton pregnancy. C, Serum hCG is lower only in pregnancies during miscarriage if the time elapsed between the blood test and diagnosis of miscarriage is less than 21 days. D, Scatterplot of serum hCG vs time elapsed until the diagnosis of miscarriage during asymptomatic singleton pregnancy. *, *P* < .05; ***, *P* < .001.

Ultrasonography confirmed fetal viability in 13 of 50 pregnancies who later miscarried, an average of 34.8 ± 6.8 days (range 2–85 d) after the kisspeptin measurement. We investigated in more detail the length of time after the kisspeptin and hCG measurements, when patients were diagnosed with miscarriage (Figure 3).

#### Kisspeptin

Compared with unaffected pregnancies, kisspeptin was significantly lower in women who experienced miscarriage when compared with unaffected pregnancies (Figure 3A); the lowest levels were observed in patients miscarrying during the first week after the blood measurement (kisspeptin in picomoles per liter: 2250 ± 1045, no miscarriage; 195 ± 116, miscarriage < 7 d, *P* < .001 vs no miscarriage). Intermediate values were obtained in patients miscarrying between 7 and 21days after the blood test (kisspeptin in picomoles per liter: 709 ± 75,0 miscarriage 7–21 d, *P* < .001 vs no miscarriage and *P* < .001 vs miscarriage < 7 d). Kisspeptin levels in women miscarrying longer than 21 days after the blood test were higher than the other miscarriage groups, but significantly lower than the group that did not miscarry (kisspeptin in picomoles per liter: 951 ± 716 miscarriage > 21 d, *P* < .001 vs no miscarriage) (Figure 3A). There was a significant correlation between kisspeptin and the time after the blood measurement when miscarriage was diagnosed (r^2^ = 0.23, *P* < .001) (Figure 3B).

#### Human chorionic gonadotropin

This was lower only in women who experienced miscarriage when compared with unaffected pregnancy, if the blood measurement was within 3 weeks prior to the diagnosis of miscarriage (hCG in international units per liter × 1000: 85.1 ± 39.3, no miscarriage; 17.2 ± 21.6, miscarriage < 7 d, *P* < .05, vs no miscarriage; 51.8 ± 55.6, miscarriage 7–21 d, *P* < .05, vs no miscarriage; 63.8 ± 37.0, miscarriage > 21 d, *P* = NS vs < 7 d) (Figure 3C). hCG did not correlate significantly with the time after the blood measurement that miscarriage was diagnosed (r^2^ = 0.028, *P* = NS) (Figure 3D).

### Relationship between plasma kisspeptin and miscarriage in multiple pregnancies

Thirty-two of the subjects had multiple pregnancies. We therefore investigated whether and how the observed relationship between kisspeptin and viability of pregnancy might also apply to multiple pregnancies. MoM kisspeptin was significantly higher in twin pregnancies unaffected by miscarriage when compared with singleton pregnancies unaffected by miscarriage (MoM kisspeptin: 1.06 ± 0.42, singleton, no miscarriage, 1.64 ± 0.54, twin no miscarriage, *P* < .001, vs singleton, no miscarriage; 2.09 ± 0.26, triplet, no miscarriage, *P* < .05, vs singleton, no miscarriage) (data not shown). In pregnancies uncomplicated by miscarriage, MoM hCG was significantly higher in twin pregnancy, but not triplet pregnancy, when compared with singleton pregnancy (MoM hCG: 1.08 ± 0.47, singleton, no miscarriage; 1.70 ± 0.49, twin, no miscarriage, *P* < .001, vs singleton, no miscarriage; 1.88 ± 0.27, triplet, no miscarriage, *P* = NS vs twin, no miscarriage) (data not shown). Ten of the 30 twin pregnancies and neither of the triplet pregnancies resulted in the death of a single fetus. The death of a single fetus during twin pregnancy significantly reduced MoM kisspeptin and MoM hCG to levels comparable with successful singleton pregnancy (MoM kisspeptin: 1.06 ± 0.42, singleton, no miscarriage; 1.15 ± 0.58, twin pregnancy with miscarriage of one fetus, *P* = NS vs singleton, no miscarriage) (MoM hCG: 1.08 ± 0.47, singleton, no miscarriage; 1.23 ± 0.84, *P* = NS vs singleton, no miscarriage) (data not shown).

## Discussion

Miscarriage is a tragic event, occurring most often before the sixth week of gestation due to nonviable chromosomal defects (8). Miscarriages after the sixth week of gestation are less common, but are more strongly associated with identifiable pathologies such as intrauterine infection and autoimmune disorders. It is therefore important to investigate whether simple and safe tests can be developed to identify pregnancies at high risk of miscarriage, because this could improve the quality of information given to patients, facilitate enhanced or targeted obstetric surveillance, and potentially improve obstetric outcomes. Kisspeptin has been recently identified as a hormone essential for fertility, pubertal development, and an important regulator of placental invasion. We report the results of the first large study of plasma kisspeptin levels at the booking antenatal visit. Our data suggest for the first time that plasma kisspeptin at the antenatal booking visit is dramatically reduced in asymptomatic women at a gestation of 6 weeks or longer who later experience miscarriage when compared with unaffected pregnancies.

A rising level of serum hCG measured during multiple hospital visits is currently used to assess fetal viability during early pregnancy (26). However, this approach is valid only if performed before the eighth gestational week when serum hCG levels are still rising, as our data and previous reports demonstrate (27). Furthermore, serum hCG is in general not used as a biomarker for pregnancy viability beyond the first trimester of pregnancy (28). Our data indicate that a single measurement of either serum hCG or kisspeptin at a gestation of 6 weeks or longer helps discriminate between viable and nonviable pregnancies, but plasma kisspeptin had superior diagnostic performance. In singleton pregnancies, a first-trimester kisspeptin level of less than 1463 pmol/L had an 80% sensitivity (95% CI 66%–90%) and a 75% specificity (95% CI 72%–78%) for miscarriage, and a kisspeptin level of less than 1630 pmol/L had 86% sensitivity (95% CI 73%–94%) and 70% specificity (95% CI 67%–73%) for detecting miscarriage. In comparison, a first trimester hCG level of less than 61 440 IU had a 72% sensitivity (95% CI 57.5%–84%) and 71% specificity (95% CI 68%–74%) for miscarriage. Combining the hCG and kisspeptin measurements as a predictive test for viability did not greatly improve the diagnostic accuracy achieved using kisspeptin alone, indicating that perturbation of kisspeptin secretion in our cohort of women ranging from 6 to 29 weeks of gestation was more frequently associated with miscarriage than changes in serum hCG concentration. This may be because defects in hCG secretion or regulation in pregnancy are more important earlier in fetal development, such as at implantation, whereas kisspeptin-related defects may appear later in gestation.

Kisspeptin was originally termed metastin (14), owing to its original description as a regulator of tumor metastasis and invasion into surrounding tissue. In keeping with this role, *KISS1* is expressed abundantly within the syncytiotrophoblast cells of the placenta as it invades into the uterine wall. It is therefore possible that kisspeptin regulates placental invasion and may represent a novel marker of placental function. Expression of kisspeptin is reduced in placental tissue from miscarried pregnancies when compared with gestation-matched pregnancies (21). Furthermore, case-control studies have reported that reduced plasma kisspeptin levels are observed in women with preeclampsia when compared with uncomplicated pregnancies (29). Circulating kisspeptin levels have been reported to be lower in women with threatened miscarriage (pain or bleeding during pregnancy) when compared with women with uncomplicated pregnancy (30). However these data are difficult to interpret because no correction was made for gestation, which was lower in the miscarriage cases when compared with cases without miscarriage.

Using logistic regression, we observed that plasma kisspeptin deficiency remains highly associated with miscarriage, even after correction for gestation and other clinical factors at the time of plasma kisspeptin measurement. Plasma kisspeptin was measured in the women in the cohort with multiple pregnancies during their booking antenatal visit. The mean plasma kisspeptin level was higher in twin pregnancies and highest in triplet pregnancies. However, the death of a single fetus in a twin pregnancy was associated with a mean plasma kisspeptin level that was comparable with levels measured in viable singleton pregnancies. Taken together, these findings suggest that circulating kisspeptin levels are derived solely from functioning placental tissue and directly reflect viable placental tissue volume. In keeping with this, the high plasma kisspeptin levels during pregnancy fall to low nonpregnant levels within days of delivery of the baby and passing of the placenta (18). These observations are also in keeping with the excellent performance of kisspeptin as a biomarker for miscarriage in women at a gestation of 6 weeks or longer in our study.

Our observation that the slope coefficient for correlation of kisspeptin levels with the time of gestation across the cohort of women who experienced miscarriage was significantly lower than for those who had an unaffected pregnancy suggests that a higher rate of increase in circulating plasma kisspeptin would be expected in a viable as compared with a failing pregnancy. It would be interesting therefore to examine this directly in a further study in which serial measurements of kisspeptin in individual patients would be collected. The serial measurements could then be used to derive kinetic parameters that could further enhance the diagnostic performance of the kisspeptin measurement for the assessment of fetal viability. Another possibility would be to compare absolute and kinetic parameters for both hCG and kisspeptin measurement to determine optimal sampling times and an optimal combination to maximize discrimination.

In our study women were recruited at their routine antenatal booking visit; therefore, blood samples were not collected during the earliest stages of pregnancy (ie, < 6 wk gestation). Further studies are needed to determine whether the high observed performance of kisspeptin to detect miscarriage remains true during earlier gestation. It is reassuring that follow-up data were available on fetal viability in the vast majority (ie, 99%) of subjects included in our study; however, we excluded 12 patients that were lost to follow-up who may (or may not) have suffered a miscarriage. This study was carried out in a tertiary obstetric center that provides care for women with low- and high-risk pregnancies as well as pregnancies that resulted from natural and assisted conception; therefore, the results may be generalized to similar populations of pregnant women. During the current study, samples were stored at −20°C between 10 and 31 months prior to the kisspeptin measurement. We therefore examined whether levels of plasma kisspeptin were related to the duration of storage. There was no significant difference between MoM kisspeptin levels among samples stored for 10, 27, 28, 29, 30, and 31 months in patients with singleton pregnancies that did not miscarry. Our data therefore suggest that the duration of sample storage did not significantly affect plasma kisspeptin levels.

In summary, we have performed the largest study to date, prospectively determining levels of plasma kisspeptin in a large cohort of pregnant women attending their antenatal booking visit. Our data suggest that plasma kisspeptin measurement in asymptomatic women at a gestation of 6 weeks or longer at their antenatal booking visit, either alone or in combination with hCG, may be associated with miscarriage risk.
